# Tobacco carcinogen induces tryptophan metabolism and immune suppression via induction of indoleamine 2,3-dioxygenase 1

**DOI:** 10.1038/s41392-022-01127-3

**Published:** 2022-09-07

**Authors:** Fan Liang, Gui-Zhen Wang, Yan Wang, Ya-Ning Yang, Zhe-Sheng Wen, Dong-Ni Chen, Wen-Feng Fang, Bin Zhang, Lu Yang, Chen Zhang, Si-Chong Han, Fu-Ying Yang, Di Wang, Li-Jun Liang, Zheng Wang, Yong Zhao, Chang-Li Wang, Li Zhang, Guang-Biao Zhou

**Affiliations:** 1 State Key Laboratory of Molecular Oncology, National Cancer Center, National Clinical Research Center for Cancer, Cancer Hospital, Chinese Academy of Medical Sciences and Peking Union Medical College, Beijing, China; 2 State Key Laboratory of Membrane Biology, Institute of Zoology, Chinese Academy of Sciences, University of Chinese Academy of Sciences, Beijing, China; 3 Department of Medical Oncology, National Cancer Center/National Clinical Research Center for Cancer/Cancer Hospital, Chinese Academy of Medical Sciences and Peking Union Medical College, Beijing, China; 4 State Key Laboratory of Oncology in South China, Collaborative Innovation Center for Cancer Medicine, Medical Oncology Department, Sun Yat-Sen University Cancer Center, Guangzhou, China; 5 Department of Lung Cancer, Tianjin Lung Cancer Center, National Clinical Research Center for Cancer, Key Laboratory of Cancer Prevention and Therapy, Tianjin’s Clinical Research Center for Cancer, Tianjin Medical University Cancer Institute and Hospital, Tianjin, China; 6 Department of Medical Oncology and Radiation Sickness, Peking University Third Hospital, Beijing, China

**Keywords:** Lung cancer, Cancer metabolism

## Abstract

Indoleamine 2,3-dioxygenase 1 (IDO1), the enzyme that catabolizes tryptophan (Trp) metabolism to promote regulatory T cells (Tregs) and suppress CD8^+^ T cells, is regulated by several intrinsic signaling pathways. Here, we found that tobacco smoke, a major public health concern that kills 8 million people each year worldwide, induced IDO1 in normal and malignant lung epithelial cells in vitro and in vivo. The carcinogen nicotine-derived nitrosaminoketone (NNK) was the tobacco compound that upregulated IDO1 via activation of the transcription factor c-Jun, which has a binding site for the *IDO1* promoter. The NNK receptor α7 nicotinic acetylcholine receptor (α7nAChR) was required for NNK-induced c-Jun activation and IDO1 upregulation. In A/J mice, NNK reduced CD8^+^ T cells and increased Tregs. Clinically, smoker patients with non-small-cell lung cancer (NSCLC) exhibited high IDO1 levels and low Trp/kynurenine (Kyn) ratios. In NSCLC patients, smokers with lower IDO1 responded better to anti-PD1 antibody treatment than those with higher IDO1. These data indicate that tobacco smoke induces IDO1 to catabolize Trp metabolism and immune suppression to promote carcinogenesis, and lower IDO1 might be a potential biomarker for anti-PD1 antibodies in smoker patients, whereas IDO1-high smoker patients might benefit from IDO1 inhibitors in combination with anti-PD1 antibodies.

## Introduction

Lung cancer represents the most common cause of cancer-related deaths, with 2,206,771 new cases and 1,796,144 deaths annually worldwide.^1^ This disease comprises small-cell lung cancer (SCLC; about 15% of all lung cancer) and non-small-cell lung cancer (NSCLC; about 85%), whereas NSCLC consists of lung adenocarcinoma (LUAD; 40%), lung squamous cell carcinoma (LUSC; 30%), and large cell lung cancer (LCLC; 15%). Tobacco smoke is the No. 1 risk factor for lung cancer, accounting for >80% of lung cancer deaths. Tobacco smoke exerts hazardous effects on exposed populations by inducing genomic mutations,^2,3^ epigenetic abnormalities,^4,5^ chronic cancer-promoting inflammation,^6,7^ and immune escape^8,9^ to promote lung cancer. However, the key molecules that mediate tobacco smoke-induced lung carcinogenesis remain to be identified, and the effects of tobacco on cell metabolism are still largely unknown.

Tryptophan (Trp), an indispensable amino acid that is mainly obtained from the diet, is used by eukaryotic cells to synthesize proteins and neurotransmitters/neuromodulators, and to produce several metabolites including kynurenine (Kyn).^10^ Indoleamine 2,3-dioxygenase 1 (IDO1) is a cytosolic heme-containing enzyme that functions to catalyze Trp degradation and Kyn production, and the Trp:Kyn ratio is usually used to reflect IDO1 activity.^11^ IDO1 is overexpressed in many types of cancers^12,13^ and is frequently detected in tumor and stromal cells.^14,15^ IDO1 expression level is inversely associated with overall survival time of the patients.^14^ By catalyzing Trp degradation and Kyn production, IDO1 facilitates an immunosuppressive microenvironment characterized by the accumulation of the naive T cell-derived regulatory T cells (Tregs) and the inhibition of effector T cells.^16–18^ IDO1 expression is regulated by interferons (IFNs), programmed death receptor 1 (PD1), KIT, RAS, pathogen-associated molecular patterns (PAMPs), and damage-associated molecular patterns (DAMPs) through relevant signaling pathways, such as IFN-γ/Janus kinase (JAK)/signal transducer and activator of transcription (STAT), phosphatidylinositol-3-kinase (PI3K)/protein kinase C (PKC), and nuclear factor kappa-light-chain-enhancer of activated B cells (NF-κB).^12^ However, the effects of environmental factors, the main causes of human cancers,^19,20^ on IDO1 expression have rarely been explored.

IDO1 has been regarded as an attractive drug target,^10^ but the best overall response to two IDO1 inhibitors, epacadostat^21^ and indoximod,^22^ was stable disease. To obtain maximum clinical benefits, several combinatory therapies including IDO1 inhibitors in combination with anti-PD1 antibodies (such as pembrolizumab)^10^ have been proposed. Unfortunately, the combined use of epacadostat and pembrolizumab did not improve progression-free survival of the melanoma patients in a phase III trial.^23^ These observations suggest that patient stratification is important for IDO1 inhibitor-based treatment, and elucidation of the crosstalk between IDO1 and other pathways and the mechanisms underlying IDO1 constitutive activation in cancers will be critical to development of effective therapeutic strategies.

Tobacco kills 8,000,000 people annually worldwide, including 7,000,000 deaths from direct tobacco use and 1,200,000 deaths from nonsmokers being exposed to second-hand smoke.^24^ Evidence for tobacco smoke on metabolic reprogramming has recently emerged, and metabolic reprogramming is regarded as an initiator of cigarette smoke-caused inflammatory pulmonary diseases.^25,26^ In NSCLC, IDO1 overexpression is correlated with former smokers,^27^ and a high Trp/Kyn ratio is correlated with a low IDO1 expression level in never-smoker patients,^28^ whereas coexpression of IDO1 and programmed cell death 1 ligand (PD-L1) is associated with smoking history.^29^ Furthermore, a low Trp/Kyn ratio was associated with resistance to PD1 blockade therapies in patients with NSCLC.^30^ Therefore, it is interesting to unveil the effects of tobacco smoke on IDO1 expression and Trp metabolism in NSCLC.

In this study, we tested the plasma concentrations of Trp and Kyn, compared the Trp/Kyn ratio in smoker and nonsmoker patients with NSCLC, analyzed the effects of cigarette smoke extract (CSE) and related carcinogens on IDO1 expression and investigated the underlying mechanisms. We found that tobacco smoke as well as its carcinogen 4-(N-methyl-N-nitrosamino)-1-(3-pyridyl)-1-butanone (NNK, also known as nicotine-derived nitrosaminoketone), induces Trp/Kyn imbalances and CD8^+^ T-cell repression as well as Treg expansion through the α7 nicotinic acetylcholine receptor (nAChR)-c-JUN-IDO1 cascade.

## Results

### Trp metabolism is more active in smoker patients than in nonsmoker NSCLC patients

**Fig. 1:**
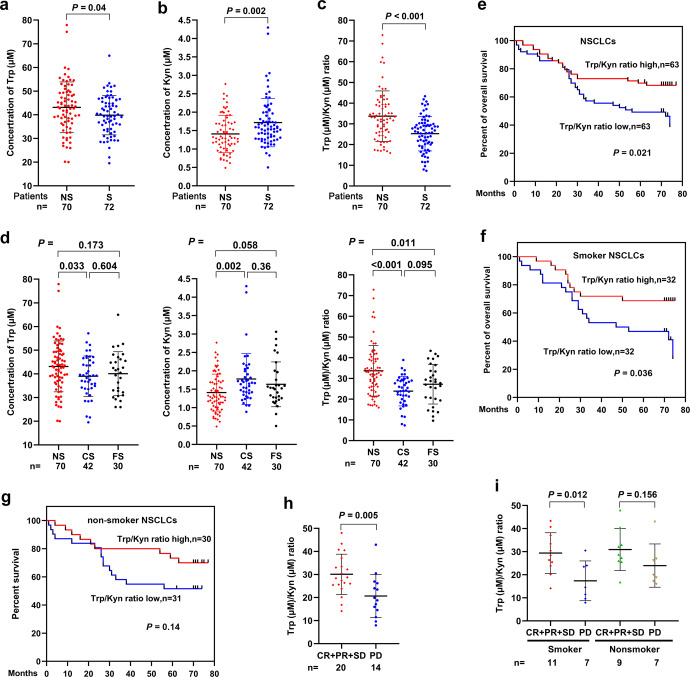
Trp metabolism is more active in smoker than in nonsmoker patients with NSCLC and is associated with poor prognosis. **a**–**c** Plasma tryptophan (Trp) concentration (**a**), kynurenine (Kyn) concentration (**b**) and Trp/Kyn ratio (**c**) in NSCLC patients. **d** Plasma concentration of Trp and Kyn and the Trp/Kyn ratio in current smoker (CS), former smoker (FS), and nonsmoker (NS) patients with NSCLC. **e**–**g** Overall survival of all patients (**e**), smoker patients (**f**) and nonsmoker patients with NSCLC (**g**). **h** The relationship between the response rate and plasma Trp/Kyn ratio in NSCLC patients treated with an anti-PD1 antibody. **i** The relationship between the response rate and the plasma Trp/Kyn ratio in smoker and nonsmoker NSCLC patients treated with an anti-PD1 antibody. *P* values, Student’s *t* test. Error bars, sd

We collected peripheral blood samples from 143 patients with NSCLC and measured the plasma concentrations of Trp and Kyn by high-pressure liquid chromatography (HPLC). The median Trp/Kyn value was used as the cut-point and Trp/Kyn ratio greater than the median is defined as high, and less than or equal to the median is defined as low. These patients were treatment-naïve, with a median age of 59 years (range, 31–78), 74 males and 68 females, and 72 smokers including 42 current smokers (having smoked at least 100 cigarettes ever and still smoked at the time of interview) and 30 former smokers (not smoking at the time of interview but had smoked at least 100 cigarettes in their life), and 70 nonsmokers (currently not smoking at the time of the interview and had smoked less than 100 cigarettes in their life; Table 1). We found that among these patients, the median Trp concentration was 41.1 (range, 19.54–77.92) μM, and the median Kyn concentration was 1.47 (range, 0.49–4.30) μM. The Trp/Kyn ratio was 28.70 (range, 7.36–72.85), and 72 (50.3%) of the 143 patients had a decreased Trp/Kyn ratio (Table 1 and Supplementary Table 1). We compared the concentrations of these two amino acids in smokers and nonsmokers and found that smokers had lower Trp (median, 39.08 μM vs. 43.55 μM, *P* = 0.02; Fig. 1a), higher Kyn (median, 1.75 μM vs. 1.20 μM, *P* = 0.002; Fig. 1b), and lower Trp/Kyn ratios (median, 21.72 vs. 34.95, *P* < 0.001; Fig. 1c) than nonsmokers. A low Trp/Kyn ratio was seen in 46 (63.9%) of the 72 smoker patients and in 25 (39.7%) of the 70 nonsmoker patients (*P* = 0.03, Table 1). Moreover, current smokers had a slightly lower plasma Trp concentration/higher Kyn concentration/lower Trp/Kyn ratio than former smokers, and the difference was not statistically significant (Fig. 1d). Moreover, current smokers showed a significantly lower Trp/Kyn ratio than nonsmokers (Fig. 1d). We further showed that among the smokers, the Trp/Kyn ratios were 25.79 (median; range, 7.36–43.34) for the 59 (81.9%) male patients, not significantly different from that (median, 26.8; range, 11.65–33.67) for the 13 (18.1%) female patients (Supplementary Table 2). Among the nonsmokers, the Trp/Kyn ratios were 30.42 (median; range, 18.81–59.45) for the 18 (25.7%) male patients, not significantly different from that (median, 33.02; range, 15.94–72.85) for the 51 (72.9%) female patients (Supplementary Table 3). These results suggested that tobacco smoking, rather than gender, is associated with low Trp/Kyn ratio in the patients.

**Table 1:** Baseline demographic characteristics of the 143 NSCLC patients

Variable	No. of cases (%)	Trp/Kyn ratio		
		High, *n* (%)	Low, *n* (%)	*P* values*
Total	143	71 (49.7)	72 (50.3)	
Age at diagnosis				0.025
<60	72 (50.3)	43 (59.7)	29 (40.3)	
≥60	70 (49)	27 (38.6)	43 (61.4)	
Unknown	1 (0.7)	1 (100)	0 (0)	
Gender				0.303
Male	74 (51.7)	33 (44.6)	41 (55.4)	
Female	68 (47.6)	37 (54.4)	31 (45.6)	
Unknown	1 (0.7)	1 (100)	0 (0)	
Smoke status				0.002
Smoker	72 (50.3)	26 (36.1)	46 (63.9)	
Nonsmoker	70 (49)	45 (64.3)	25 (35.7)	
Unknown	1 (0.7)	0 (0)	1 (100)	
Histology				0.406
LUAD	84 (58.7)	44 (52.4)	40 (47.6)	
LUSC	58 (40.6)	26 (44.8)	32 (55.2)	
Unknown	1 (0.7)	1 (100)	0 (0)	
Stage				0.021
I–II	49 (34.3)	30 (61.2)	19 (38.8)	
III–IV	65 (45.4)	24 (36.9)	41 (63.1)	
Unknown	29 (20.3)	17 (58.6)	12 (41.4)	

*P values were calculated using a two-sided Fisher’s exact test

### A lower Trp/Kyn ratio is associated with poor prognosis in patients with NSCLC

We analyzed the relationship between the Trp/Kyn ratio and the clinical outcome of the patients and found that NSCLC patients with lower Trp/Kyn ratios (*n* = 63) had a worse prognosis than those with higher Trp/Kyn ratios (*n* = 63; *P* = 0.021, Fig. 1e), consistent with previous reports.^31,32^ In smoker patients (*n* = 64), lower Trp/Kyn ratio was associated with worse prognosis (Fig. 1f); in nonsmoker patients (*n* = 61), patients with lower Trp/Kyn had a slightly (though not statistically significantly) shorter life span than those with higher Trp/Kyn ratios (Fig. 1g). We found that in 34 NSCLC patients treated with an anti-PD1 antibody, patients with lower Trp/Kyn ratios showed a poorer response (exhibiting progressive disease, PD) to the drug than those with higher Trp/Kyn ratios [obtaining complete remission (CR), partial response (PR) or stable disease (SD); Fig. 1h]. In smokers with NSCLC, patients with higher Trp/Kyn ratios showed a better response to the anti-PD1 antibody than those with lower Trp/Kyn ratios (Fig. 1i).

We also analyzed the potential effects of the age (Supplementary Fig. 1a), gender (Supplementary Fig. 1b), smoking (Supplementary Fig. 1c) and stage (Supplementary Fig. 1d) on the prognosis of the patients, and found that only Stage had a significant effect on the outcome, in that patients at an early stage (Stage I and II, *n* = 49) had a much more favorable prognosis than patients at a late stage (Stage III and IV, *n* = 58; Supplementary Fig. 1d). However, Trp/Kyn ratio did not show a significant effect on prognosis of patients at an early (Supplementary Fig. 1e) or late stage (Supplementary Fig. 1f), possibly due to the small sample size of each group.

### Tobacco smoke and carcinogen NNK promote Trp metabolism

**Fig. 2:**
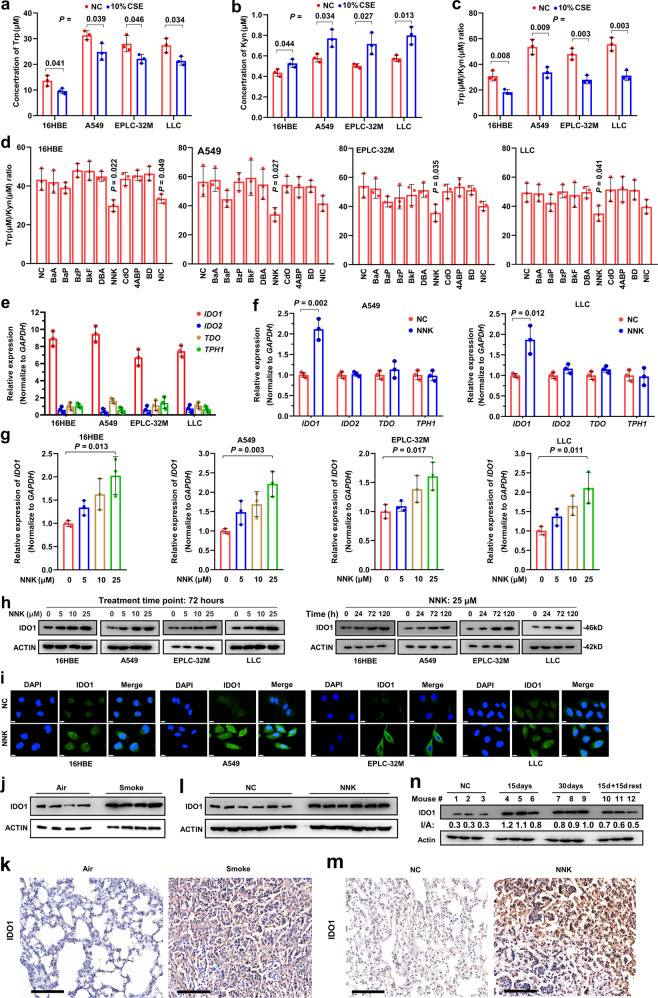
NNK causes the imbalance of Trp/Kyn through IDO1. **a**–**c** Trp concentration (**a**), Kyn concentration (**b**), and Trp/Kyn ratio (**c**) in supernatants of 16HBE, A549, EPLC-32M, and LLC cells treated with 10% cigarette smoke extract (CSE) for 72 h. **d** Trp/Kyn ratio in supernatants of 16HBE, A549, EPLC-32M, and LLC cells treated with ten tobacco compounds at 25 μM for 72 h. **e** The expression of four Trp-metabolizing enzymes in the cells. **f** The changes in the expression of Trp-metabolizing enzymes in A549 and LLC cells after treatment with NNK at 25 μM for 72 h. **g** *IDO1* expression in the cells treated with NNK at indicated concentrations for 72 h was detected by quantitative reverse transcription-polymerase chain reaction (qRT-PCR). **h** The cells were treated with NNK at indicated protocols, lysed, and subjected to western blot assays. **i** IDO1 expression in the cells treated with NNK at 25 μM for 72 h was detected by immunofluorescence assays. **j**, **k** The A/J mice were exposed to tobacco smoke for 180 days and sacrificed, and lung tissues were subjected to western blot (**j**) and immunohistochemistry (**k**) assays to detect the expression of IDO1. Scale bar = 100 μm. **l**, **m** The A/J mice were treated with NNK at 50 mg/kg/day for 90 days and sacrificed, and IDO1 expression in lung tissues was detected by western blot (**l**) and immunohistochemistry (**m**). Scale bar = 100 μm. **n** The A/J mice were treated with NNK for 15 to 30 days, or treated with NNK for 15 day and then stopped treatment for additional 15 days, sacrificed, and IDO1 expression in lung tissues was detected by western blot. *P* values, Student’s *t* test. Error bars, sd

The above data suggest that tobacco smoke may induce Trp metabolism and immunosuppression in NSCLC. To test this hypothesis, CSE was prepared^33^ and used to treat the human normal lung epithelial cell line 16HBE, the LUAD cell line A549, the LUSC cell line EPLC-32M, and the murine LUAD cell line LLC. We found that CSE at a final concentration of 10% was able to significantly reduce Trp (Fig. 2a) and increase the Kyn concentration (Fig. 2b) and decrease the Trp/Kyn ratio (Fig. 2c) in the supernatant of the cells compared to treatment with vehicle control. To identify the key carcinogen responsible for the effects of tobacco smoke on Trp metabolism, the tobacco compounds 4-aminobiphenyl (ABP), benzo[a]anthracene (BaA), benzo(a)pyrene (BaP), 1,3-butadiene (BD), benzyl 4-hydroxybenzoate (BzP), benzo[k]fluoranthene (BkF), cadmium oxide (CdO), dibenzo[a,h]anthracene (DbA), nicotine (NIC), and NNK were used to treat the cells, the Trp and Kyn concentrations of the supernatants were analyzed, and the Trp/Kyn ratio was calculated. We found that among these compounds, NNK significantly reduced the Trp/Kyn ratios in the cells (Fig. 2d). Nicotine also reduced Trp/Kyn ratios in the cells, though the effects were weaker than that of NNK (Fig. 2d).

### NNK induces IDO1 in lung epithelial cells in vitro and in vivo

IDO1, IDO2, tryptophan 2,3-dioxygenase (TDO), and tryptophan hydroxylase 1 (TPH1) play important roles in Trp metabolism.^10^ We tested the expression of these genes in 16HBE, A549, EPLC-32M, and LLC cells and found that *IDO1* expression was high, whereas *IDO2*, *TDO*, and *TPH1* expression was low in the cells (Fig. 2e). We then tested the effects of NNK on these genes and found that treatment with NNK at 25 µM for 72 h significantly upregulated *IDO1* but not *IDO2*, *TDO* or *TPH1* in A549 and LLC cells (Fig. 2f). NNK upregulated *IDO1* in a dose-dependent manner in the four cell lines (Fig. 2g). At protein level, NNK upregulated IDO1 in 16HBE, A549, EPLC-32M, and LLC cells in a dose- and time-dependent manner (Fig. 2h). Immunofluorescence analysis confirmed that IDO1 mainly localized in the cytoplasm and that NNK upregulated IDO1 in the four cell lines (Fig. 2i).

We tested the effects of tobacco smoke and NNK on IDO1 in vivo. To do this, the A/J mice were exposed to cigarette smoke (12 cigarettes per day, 5 days per week for 24 weeks)^9^ or NNK (at a dose of 50 mg/kg twice a week for 5 weeks)^6^ and sacrificed, and the lung tissues were harvested to evaluate the expression of IDO1. By western blot assay, we found that exposure to tobacco smoke markedly induced upregulation of IDO1 compared to that in lung tissues of mice exposed to fresh air (Fig. 2j). These results were confirmed by IHC assay (Fig. 2k). IDO1 was also upregulated in lung tissues of the mice treated with NNK (Fig. 2l, m). In addition, we found that the expression of IDO1 was upregulated by treatment with NNK at 50 mg/kg/day for 15–30 days (Fig. 2n). Unexpectedly, in mice treated with NNK at 50 mg/kg/day for 15 days and then rested for 15 days, the expression of IDO1 was still higher than that in mice treated with vehicle control (Fig. 2n), in consistent with the observations that former smokers had lower Trp/Kyn ratios than nonsmokers (Fig. 1d).

### IDO1 is high in smokers with NSCLC and is associated with a poor response to PD1 blockade

**Fig. 3:**
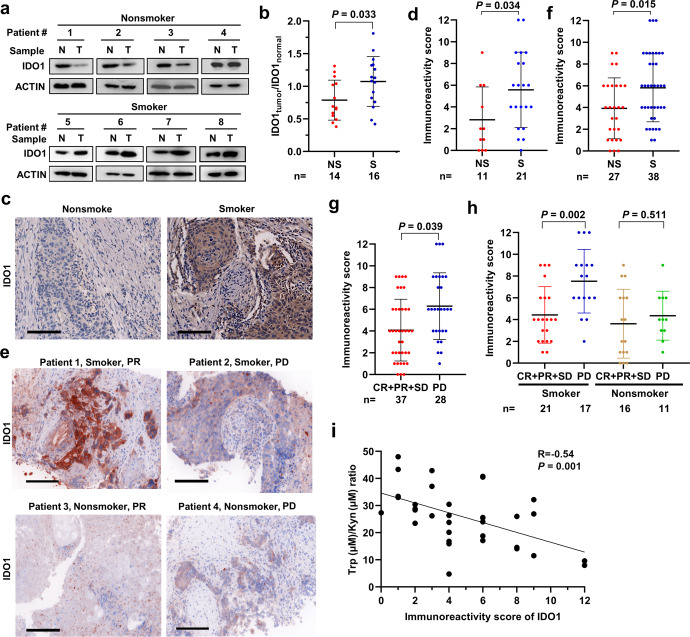
Tobacco smoke is associated with higher IDO1 expression and worse response to anti-PD1 antibody in NSCLC patients. **a**–**d** Western blot (**a**, **b**) and immunohistochemistry (**c**, **d**) assays were conducted to detect the expression of IDO1 in lung tissues of smoker and nonsmoker patients with NSCLC. Densitometry analysis of western blot bands was performed, and the ratio of IDO1 in tumors (IDO1_tumor_) to IDO1 in normal tissues (IDO1_normal_) was calculated (**b**). The immunoreactivity score is shown (**d**). Scale bar = 100 μm. **e**–**i** IDO1 immunohistochemistry staining results of lung tissues of NSCLC patients treated with the anti-PD1 antibody pembrolizumab. Scale bar = 100 μm (**e**). Immunoreactivity scores of smoker and nonsmoker patients (**f**) and of those who achieved complete remission (CR), partial remission (PR), stable disease (SD), or progressive disease (PD) are shown (**g**, **h**). The correlation between the Trp/Kyn ratio and the IDO1 immunoreactivity score is shown (**i**). *P* values, Student’s *t* test. Error bars, sd

We tested the expression of IDO1 in smoker and nonsmoker patients by western blot assay using lysates of tumor-normal paired lung tissues and found that IDO1 in normal lung tissues was higher than that in tumor samples of the nonsmoker patients (Fig. 3a). However, lung tumor samples from smoker patients had higher IDO1 than paired counterpart normal lung tissues (Fig. 3a). Densitometry analysis of western blot bands confirmed that smoker patients had higher values of IDO1 in tumor tissue (IDO1_tumor_) to IDO1 in normal tissue (IDO1_normal_) ratio than nonsmoker patients (Fig. 3b). The IHC assay showed that tumor samples of smoker patients had higher levels of IDO1 expression (Fig. 3c) and higher immunoreactivity score (IRS) (Fig. 3d) than those of nonsmoker patients.

We next explored the association between IDO1 expression and the response to pembrolizumab in 65 patients (Table 2) and found that IDO1 was higher in smokers than in nonsmokers (Fig. 3e, f). In this cohort, patients who achieved CR, PR, or SD had lower IDO1 expression than patients who obtained PD, with median IRSs of 4 (range, 0–9) and 6 (range, 1–12) for the two groups, respectively (Fig. 3g). In smokers with NSCLC, patients who obtained CR/PR/SD had a significantly lower IDO1 IRS (median: 4, range: 1–9) than those who developed PD (median: 8, range: 2–12; Fig. 3h). In nonsmoker patients treated with pembrolizumab, IDO1 in responders was not significantly different to that in nonresponders (Fig. 3h). In addition, we found that IDO1 IRS was inversely associated with the Trp/Kyn ratio (Fig. 3i), in consistent with the role of IDO1 in Trp metabolism.

**Table 2:** Baseline demographic characteristics of the 65 NSCLC patients treated with an anti-PD1 antibody

Variable	No. of cases (%)	IDO1 expression		
		High, *n* (%)	Low, *n* (%)	*P* values*
Total	65	44 (67.7)	21 (32.3)	
Age at diagnosis				0.605
≤60	37 (56.9)	24 (64.9)	13 (35.1)	
>60	28 (43.1)	20 (71.4)	8 (28.6)	
Sex				0.261
Male	44 (67.7)	32 (72.7)	12 (27.3)	
Female	21 (32.3)	12 (57.1)	9 (42.9)	
Smoke status				0.01
Smoker	38 (58.5)	30 (78.9)	8 (21.1)	
Nonsmoker	27 (41.7)	14 (51.9)	13 (48.1)	
Histology				0.014
LUAD	37 (56.9)	20 (54.1)	17 (45.9)	
LUSC	26 (40)	23 (88.5)	3 (11.5)	
Unknown	2 (3.1)	1 (50)	1 (50)	
Stage				0.331
II–III	8 (12.3)	7 (87.5)	1 (12.5)	
IV	56 (86.2)	36 (64.3)	20 (35.7)	
Unknown	1 (1.5)	1 (100)	0 (0)	

*P values were calculated using a two-sided Fisher’s exact test

### α7nAChR mediates NNK-induced IDO1 expression

**Fig. 4:**
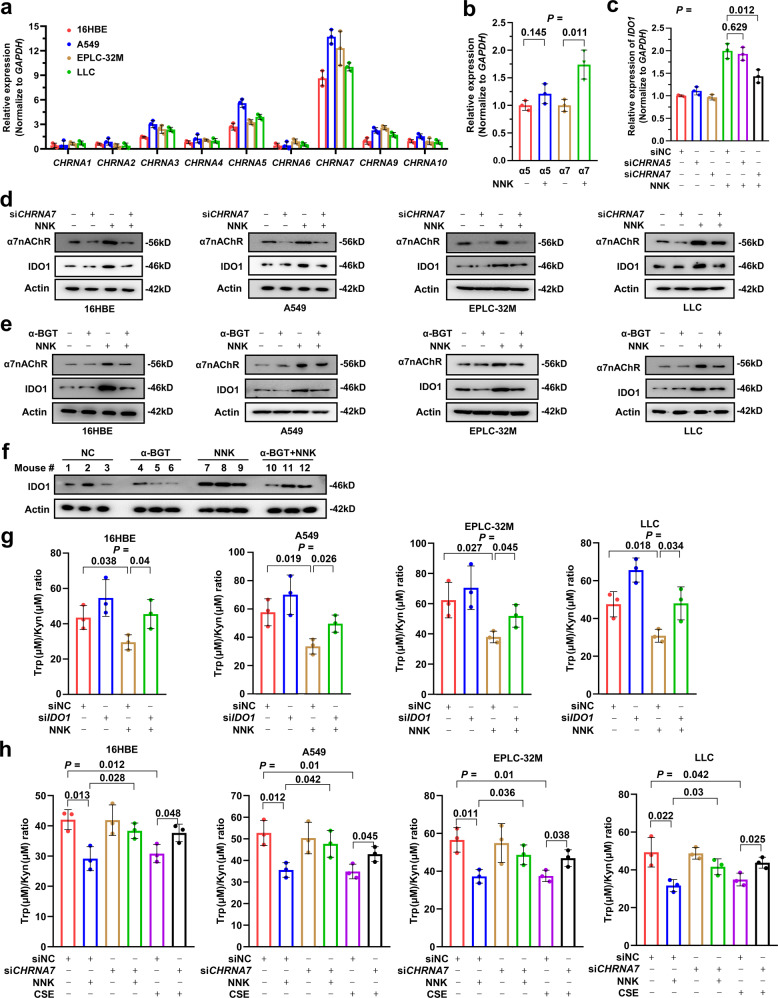
α7nAChR is required for NNK-induced IDO1 upregulation and Trp metabolism. **a** The expression of the nine NNK receptors in 16HBE, A549, EPLC-32M and LLC cells. **b** The expression of α5nAChR- and α7nAChR-coding genes in A549 cells treated with 25 μM NNK for 72 h. **c** A549 cells were transfected with or without si*CHRNA5* or si*CHRNA7* and treated with 25 μM NNK for 72 h, and the expression of *IDO1* was detected by qRT-PCR. **d**, **e** The cells were pretransfected with si*CHRNA7* (**d**) or pretreated with α-BGT (**e**), followed by treatment with 25 μM NNK for 72 h. The cells were then lysed and subjected to western blotting using the indicated antibodies. **f** The A/J mice were treated with NNK and/or α-BGT for 90 days and sacrificed, and lung tissues were collected for western blot analysis. **g** The cells were transfected with si*IDO1* and treated with 25 μM NNK for 72 h, and the Trp/Kyn ratio was tested. **h** The cells were transfected with si*CHRNA7* and treated with 25 μM NNK or 10% CSE for 72 h, and the Trp/Kyn ratio was tested. *P* values, Student’s *t* test. Error bars, sd

The carcinogenic action of NNK is known to occur through mutagenic effects and binding to its receptor nicotinic acetylcholine receptors (nAChR), which consist of nine α-subunits and four β-subunits.^34^ We tested the expression of the genes encoding nAChRs in 16HBE, A549, EPLC32M and LLC cells and found that the expression of *CHRNA7* (encoding α7nAChR) and *CHRNA5* (encoding α5nAChR) was relatively high in these cells (Fig. 4a). In A549 cells upon NNK treatment, only the α7nAChR gene was significantly upregulated (Fig. 4b). Moreover, silencing *CHRNA7* by small interfering RNA (siRNA, si*CHRNA7*) significantly inhibited NNK-induced upregulation of *IDO1*, whereas si*CHRNA5* treatment did not (Fig. 4c). In the four lines, NNK induced the upregulation of α7nAChR and IDO1, which was attenuated by si*CHRNA7* (Fig. 4d). Consistently, the specific α7nAChR inhibitor α-bungarotoxin (α-BGT) also inhibited NNK-induced upregulation of IDO1 (Fig. 4e). We further tested the effect of NNK on IDO1 in vivo by treating the mice with NNK and/or α-BGT for 3 months, at which time point no tumor was detected. We found that while NNK induced the upregulation of IDO1, α-BGT markedly inhibited this effect in the lung tissues of the mice (Fig. 4f).

We next tested the effects of IDO1 and α7nAChR on NNK-induced Trp metabolism in 16HBE, A549, EPLC32M and LLC cells and found that NNK treatment led to a decrease in the Trp/Kyn ratio, whereas silencing *IDO1* by pretreatment of the cells with si*IDO1* substantially inhibited the NNK-induced decrease in the Trp/Kyn ratio (Fig. 4g). Furthermore, we found that while silencing α7nAChR by si*CHRNA7* did not affect the Trp/Kyn ratio or Trp and Kyn concentrations, pretreatment of the cells with si*CHRNA7* significantly antagonized the NNK- and CSE-induced reduction of the Trp/Kyn ratio (Fig. 4h). These results suggested that the α7nAChR-IDO1 axis mediates tobacco smoke-induced Trp imbalance.

### c-Jun mediates NNK/α7nAChR-induced upregulation of IDO1

**Fig. 5:**
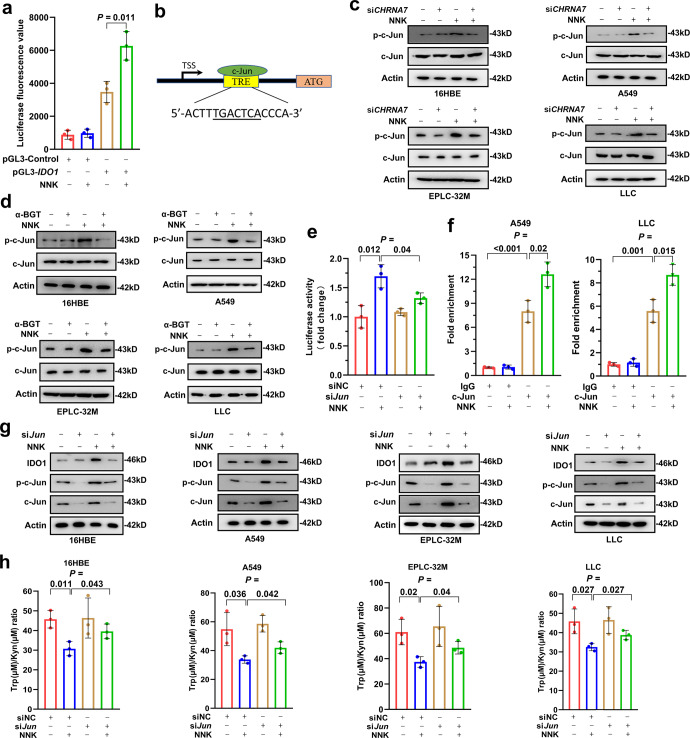
NNK upregulates IDO1 expression and promotes Trp metabolism in an α7nAChR/c-Jun-dependent manner. **a** Luciferase activity driven by the *IDO1* promoter in A549 cells treated with 25 μM NNK for 48 h. **b** c-Jun binding site in *the IDO1* promoter. TSS, transcription start site. **c**, **d** The cells were transfected with si*CHRNA7* (**c**) or pretreated with α-BGT (**d**) and then treated with 25 μM NNK for 72 h. The cells were lysed for western blotting using the indicated antibodies. **e** A549 cells expressing pGL-*IDO1* promoter-luciferase were transfected with si-*c-Jun*, treated with 25 μM NNK for 72 h, and lysed for analysis of luciferase activity. **f** A549 and LLC cells were treated with or without NNK and lysed, chromatin immunoprecipitation assays were performed using IgG or an anti-c-Jun antibody, and the precipitated DNA was used to detect the expression of *IDO1* by qRT-PCR. **g** The cells were transfected with si*c-Jun*, treated with 25 μM NNK for 72 h, lysed and subjected to western blotting using indicated antibodies. **h** The cells were transfected with si*c-Jun* and treated with 25 μM NNK for 72 h, the concentrations of Trp and Kyn in the supernatants were measured, and the Trp/Kyn ratio was calculated. *P* values, Student’s *t* test. Error bars, sd

The above results indicated that α7nAChR plays a critical role in NNK-induced IDO1 upregulation. We hypothesized that α7nAChR-associated transcription factor (TF) may mediate NNK-α7nAChR-induced IDO1 upregulation. Indeed, in A549 cells that were transfected with plasmids containing luciferase driven by *IDO1* promoter (−1800 to −30 bp upstream of ATG) (pGL-*IDO1*-luciferase), NNK treatment significantly increased the luciferase activity of the cells (Fig. 5a), supporting the above hypothesis. We therefore screened for TFs that could regulate *IDO1* expression using the online tool PROMO,^35,36^ a virtual laboratory for the study of TF binding sites in DNA sequences. Interestingly, we found that the sequence of *IDO1* has potential binding sites for 19 TFs in humans (such as C/EBPβ, c-Jun, p53, STAT4, XBP1, and YY1) and 9 TFs in mouse (such as C/EBPα, c-Jun, c-Fos, HOXA5, and YY1), and c-Jun, C/EBPβ and YY1 were found in both human and mouse (Supplementary Table 4). We then searched the literature to identify those TFs associated with NNK and/or α7nAChR and found that c-Jun, which has an *IDO1* binding site (Fig. 5b), is reported to be upregulated by nicotine,^37^ a tobacco compound from which NNK is derived.^38^

We tested the effect of NNK on c-Jun and found that NNK could upregulate the expression of phosphorylated c-Jun (p-c-Jun, the active form of c-Jun) but did not perturb the total level of c-Jun (Fig. 5c). In 16HBE, A549, EPLC-32M and LLC cells, knockdown of α7nAChR by si*CHRNA7* substantially inhibited c-Jun activation induced by NNK (Fig. 5c). Consistently, treatment of the cells with α-BGT markedly suppressed p-c-Jun upregulation induced by NNK (Fig. 5d).

We tested whether c-Jun is the main TF that mediates NNK-induced upregulation of *IDO1* in A549 cells expressing pGL3-*IDO1* promoter-luciferase and found that si*c-Jun* significantly inhibited the increase in luciferase activity induced by NNK treatment (Fig. 5e). To determine whether c-Jun could bind the *IDO1* promoter, a chromatin immunoprecipitation (ChIP) assay was conducted, and the expression level of *IDO1* was detected by qRT-PCR. We found that c-Jun could bind to the promoter of *IDO1*, and the binding efficiency was significantly increased after NNK treatment (Fig. 5f). Both C/EBPβ and YY1 could also bind the promoter of *IDO1*, but the binding efficiency could not significantly increase after NNK treatment (Supplementary Fig. 2a). By Western blotting, we found that knocking down *c-Jun*, but not C/EBPβ or YY1, inhibited the increase in IDO1 induced by NNK in the cells (Fig. 5g, Supplementary Fig. 2b). These results suggested that c-Jun is the main TF that mediates the NNK-induced increase in *IDO1* expression. We also found that knockdown of *c-Jun* inhibited the decrease in the Trp/Kyn ratio in the cell culture supernatant caused by NNK in the cells (Fig. 5h). These results indicated that NNK induces IDO1 upregulation through α7nAChR to activate c-Jun.

### NNK induces an immunosuppressive microenvironment via IDO1

**Fig. 6:**
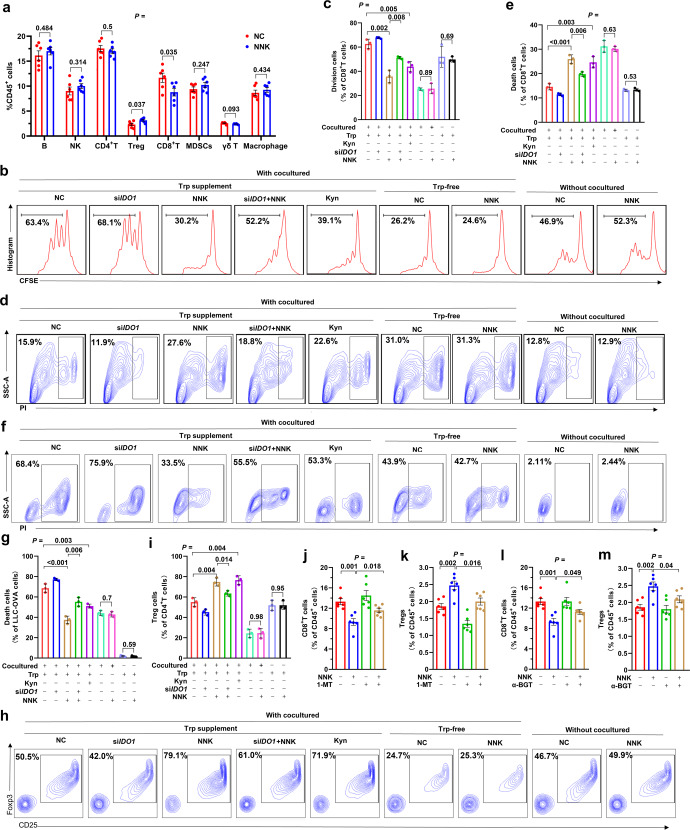
NNK promotes immunosuppression through IDO1-mediated Trp metabolism. **a** A/J mice were treated with NNK for 90 days and sacrificed, and CD45^+^ cells were isolated from lung tissues and analyzed by flow cytometry. **b**–**e** CD8^+^ T cells were harvested from C57 mice, cultured alone or in the presence of si*IDO1*-transfected LLC cells in Trp-free or Trp normal medium. The cells were treated with NNK at 25 μM for 72 h. Cell division was evaluated by cytometric analysis of 5-(and-6)-carboxyfluorescein diacetate succinimidyl ester (CFSE) (**b**, **c**), and cell death was assessed by propidium iodide (PI) and flow cytometry assay (**d**, **e**). **f**, **g** The death rate of si*IDO1*-transfected LLC-OVA cells cultured alone or cocultured with OT-1 CD8^+^ T cells in Trp-free or Trp normal medium in the absence or presence of NNK at 25 μM for 48 h. **h**, **i** CD4^+^ T cells were harvested from C57 mice and cultured in the absence or presence of si*IDO1*-transfected LLC cells in Trp-free or Trp normal medium. The cells were treated with NNK at 25 μM for 72 h. The percentage of Tregs in CD4^+^ cells was assayed by flow cytometry. **j**, **k** A/J mice were treated with NNK and/or IDO1 inhibitor 1-methyl-L-tryptophan (1-MT) for 90 days and sacrificed, and CD8^+^ T cells (**j**) and Tregs (**k**) in lung tissues were analyzed by flow cytometry. **l**, **m** A/J mice were treated with NNK and/or α-BGT for 90 days and sacrificed, and CD8^+^ T cells (**l**) and Tregs (**m**) in lung tissue were analyzed by flow cytometry. *P* values, Student’s *t* test. Error bars, sd

We analyzed the effects of NNK on immune cells in the lung tissues of mice treated with this carcinogen or vehicle control by flow cytometry analysis of CD45^+^ cells that were harvested from the mice, and found that NNK treatment significantly reduced CD8^+^ T cells and increased Treg cells but did not affect the total number and proportion of B cells, natural killer (NK) cells, CD4^+^ T cells, myeloid-derived suppressor cells (MDSCs), γδ T cells, or macrophages (Fig. 6a, Supplementary Fig. 3a). To determine the role of IDO1-associated Trp metabolism in NNK-induced suppression of CD8^+^ T cells, CD8^+^ T cells were isolated from the spleen and lymph nodes (LNs) of C57BL/6 mice using PE-CY7-labeled anti-CD8 antibody by flow cytometric cell sorting, labeled by 5-(and-6)-carboxyfluorescein diacetate succinimidyl ester (CFSE), and cultured alone or cocultured with si*IDO1*-treated LLC cells (2:1) in RPMI 1640 medium with 10% FBS, 1 ng/ml IL-2 and activation by the precoated 5 μg/ml anti-CD3 antibody and 5 μg/ml CD28 antibody. The proliferation of the cells was assessed by flow cytometry for CFSE fluorescence 3 days later, and Kyn was used as a control. We found that when cultured alone in the presence of Trp, NNK did not affect the proliferation of the cells (Fig. 6b, c). In the co-culture system, Kyn inhibited CD8^+^ T-cell proliferation; in this co-culture and absence of Trp conditions, NNK did not affect CD8^+^ T-cell proliferation compared to the vehicle control. In the presence of Trp and LLC cells, NNK substantially inhibited the proliferation of CD8^+^ T cells (Fig. 6b, c). Under this normal co-culture and Trp condition, NNK treatment suppressed CD8^+^ T-cell proliferation, which could be attenuated by si*IDO1*-mediated IDO1 silencing in LLC cells (Fig. 6b, c). In the co-culture system, starvation of Trp and treatment with Kyn led to death of CD8^+^ T cells (Fig. 6d, e). In the presence of Trp and LLC cells, NNK-induced cell death of CD8^+^ T cells; in this co-culture and Trp normal condition, si*IDO1* treatment of LLC cells suppressed cell death, whereas NNK promoted death of CD8^+^ T cells (Fig. 6d, e). These results suggested that NNK exerts inhibitory effects on CD8^+^ T cells in an IDO1-dependent manner.

The effects of NNK on the cytotoxicity of CD8^+^ T cells were evaluated in Ovalbumin (OVA)-specific CD8^+^ T cells isolated from the spleen and LNs of OT-1 transgenic C57BL/6 mice. The isolated OT-1 CD8^+^T cells were cultured in RPMI 1640 medium supplemented with 1 ng/ml IL-2, 5 μg/ml anti-CD3 antibody and 2 μg/ml OVA peptide. The LLC-OVA cells were labeled with CFSE and cultured alone or cocultured with OT-1 transgenic CD8^+^ T cells (1:10 ratio), and PI was added 48 h later to detect cell death. We found that in this coculture system, Kyn inhibited LLC-OVA cell death; without CD8^+^ T cells or CD8^+^ T cells were added but Trp was absent, NNK did not induce LLC-OVA cell death; in the coculture system supplemented with Trp, LLC-OVA cell death was obvious (65.4%), and NNK was able to reduce cell death (death rate was 35.2%) (Fig. 6f, g). In the presence of CD8^+^ T cells and Trp, si*IDO1* treatment increased LLC-OVA cell death, while NNK reduced LLC-OVA cell death (Fig. 6f, g). These results suggested that NNK might affect the cytotoxicity of CD8^+^ T cells via IDO1-mediated Trp metabolism.

We tested the effects of NNK on Treg differentiation. To this end, naïve CD4^+^ T cells were isolated from the spleen and LNs of C57BL/6 mice by magnetic negative sorting, cultured alone or cocultured with LLC cells (2:1) and activated by precoated 5 μg/ml anti-CD3 antibody and 5 μg/ml CD28 antibody. Seventy-two hours later, the proportion of CD25^+^/Foxp3^+^ cells was analyzed. We found that when cultured alone, NNK did not affect Treg differentiation. When cocultured with LLC cells, Kyn and NNK increased the proportion of CD25^+^/Foxp3^+^ Tregs (Fig. 6h, i). Knocking down *IDO1* in LLC cells before co-culture reversed the increase in Tregs. When cocultured in Trp-free medium, NNK had no significant effect on the Treg proportion (Fig. 6h, i).

**Fig. 7:**
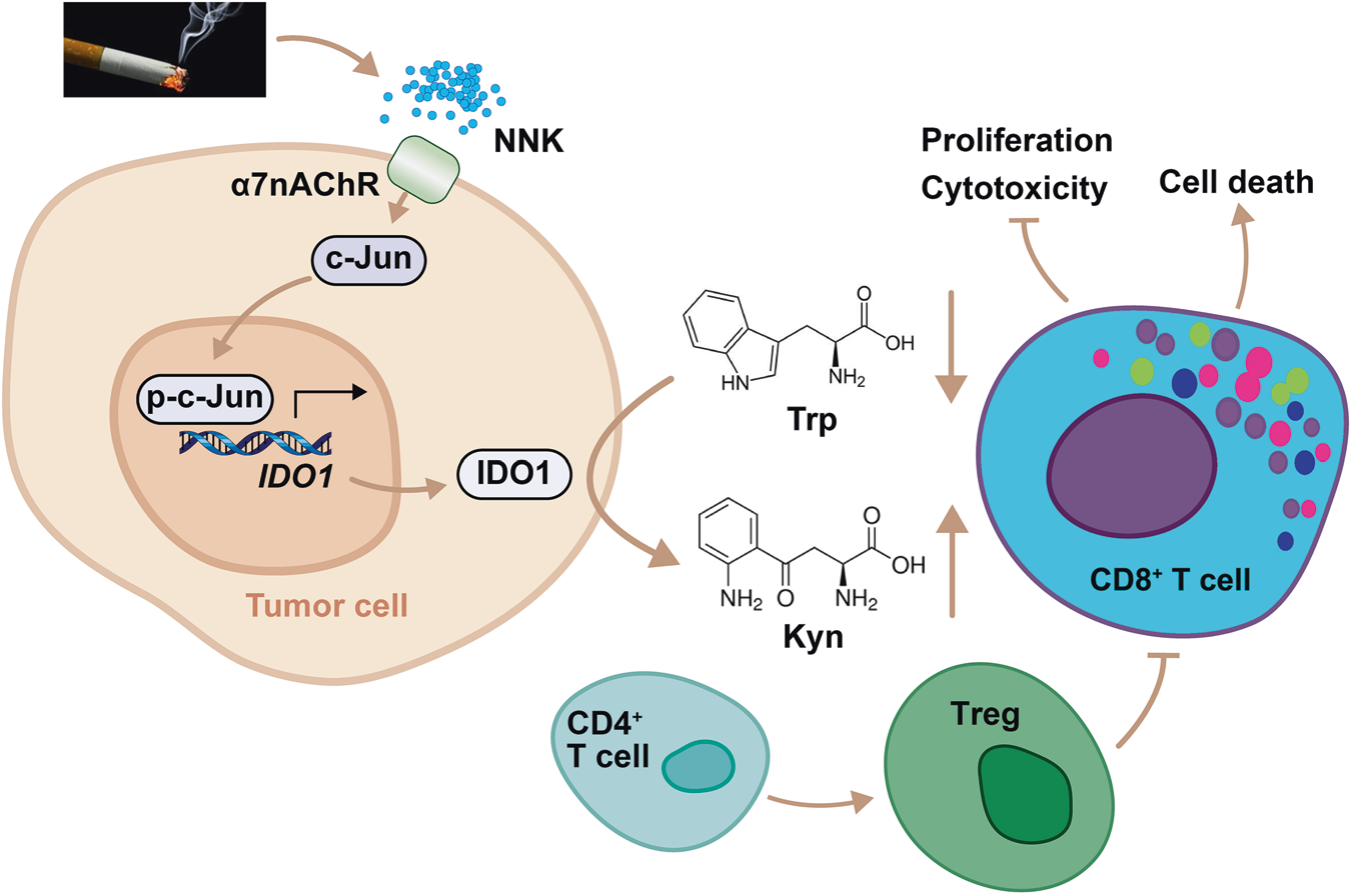
Mechanisms of action of tobacco smoke in promotion of tryptophan catabolism and immunosuppressive microenvironment. Tobacco smoke activates c-Jun through NNK-α7nAChR axis, leading to upregulation of IDO1 in tumor cells. IDO1 triggers catabolism of tryptophan and production of kynurenine, resulting in suppression of CD8^+^ T cells and accumulation of Tregs. These effects promote environmental lung carcinogenesis

We further tested the role of IDO1 in CD8^+^ T cells and Tregs in vivo by treating the mice with NNK and/or the IDO1 inhibitor 1-methyl-L-tryptophan (1-MT) for 3 months and found that while NNK significantly reduced CD8^+^ T cells (Fig. 6j, Supplementary Fig. 3b) and increased Tregs (Fig. 6k), 1-MT significantly attenuated these effects (Fig. 6j, k). In addition, α-BGT also significantly antagonized NNK-induced CD8^+^ T-cell suppression (Fig. 6l, Supplementary Fig. 3c) and Treg proliferation (Fig. 6m, Supplementary Fig. 3c). The above results suggested that the α7nAChR-IDO1 cascade is required for NNK-induced immune suppression in the lungs (Fig. 7).

## Discussion

Metabolic reprogramming is a hallmark of cancer.^39,40^ Several lines of evidence have recently emerged indicating that tobacco smoke can trigger metabolic reprogramming. For example, proteomic analysis of lung epithelial cells exposed to cigarette smoke shows abnormal oxidative phosphorylation and increase in enzymes for the tricarboxylic acid cycle.^26^ In macrophages, CSE exposure inhibits oxidative phosphorylation and induces glycolysis and the generation of reactive oxygen species.^41^ However, tobacco smoke-related metabolic abnormalities in lung cancer remain to be revealed. Here, we showed that tobacco smoke could enhance Trp metabolism in human lung epithelial cells and in mice through NNK-induced upregulation of IDO1. The results in cellular and animal models were seen in NSCLC patients, in that smoker patients had higher IDO1 expression levels and lower Trp/Kyn ratios than nonsmoker patients. These results indicated that the abnormal Trp metabolism in NSCLC may be the result of tobacco exposure, and the causes of other metabolic dysfunctions in cancer warrant further investigation. However, 26 (36.1%) of 72 smoker patients had a high Trp/Kyn ratio, possibly attributed to their metabolic activity for tobacco carcinogens and differences in the composition of tobacco they smoked. In addition, the upregulation of IDO1 by NNK provides a new example of an environment-gene interaction in lung cancer.

Avoiding immune destruction is another hallmark of cancer.^40^ In NSCLC, overexpressed PD-L1 on cancer cells binds its receptor PD1 and inhibits T-cell activation and cytokine production.^42^ Blockade of this interaction exerts significant therapeutic efficacy in NSCLC,^43–45^ and current or former smoking status was associated with an increased response to treatment.^44,45^ We investigated the underlying mechanism and found that tobacco smoke induces upregulation of PD-L1 via carcinogen BaP-induced activation of aryl hydrocarbon receptor (AhR), which binds the *PD-L1* promoter and controls its transcription. Moreover, patients expressing high levels of AhR in tumor tissues responded better to pembrolizumab than patients with lower levels of AhR.^9^ In this study, we found that tobacco smoke upregulated IDO1 in lung epithelial cells through NNK-induced α7AChR-c-Jun signaling pathway. IDO1 in tumor cells, the dominant cell type in NSCLC, metabolizes Trp and reduces its availability for immune cells, leading to suppression of CD8^+^ T cells and expansion of Tregs. In addition, Kyn is able to induce and activate AhR and thereby upregulates PD1 expression in CD8^+^ T cells.^46^ These results indicate that tobacco smoke may induce a tumor immunosuppressive microenvironment via different carcinogens and different mechanisms, resulting in heterogeneous immune profiles of the patients and diverse responses to immunotherapies. These data also suggested that the imbalances in Trp metabolism and T-cell repression may originate from tobacco smoke, and the immunological heterogeneity of the patients may have its roots in exposure to environmental risk factors.

nAChR, which was identified one hundred years ago and represented the first purified ion channel,^47^ consists of nine alpha subunits (α1–7, α9, α10), four beta subunits (β1–4), and one delta (δ), one gamma (γ) and one epsilon (ε) subunits.^34^ nAChRs are widely distributed in diverse neuronal and nonneuronal tissues, including the respiratory tract. We tested the expression of the nine alpha subunits in 16HBE, A549, EPLC-32M, and LLC cells and found that α7nAChR expression was the highest, consistent with previous observations that human bronchial epithelial and endothelial cells express alpha7.^48^ In NSCLC, α7nAChR was upregulated and was associated with unfavorable prognosis.^49^ α7nAChR promotes cell proliferation, angiogenesis, and metastasis and mediates drug resistance via signaling pathways, including c-Jun, GATA4/6, PI3K-Akt, MEK/ERK, and FGF2.^50^ We found that α7nAChR was upregulated by NNK and was required for NNK-induced IDO1 expression, and inhibition of α7nAChR by siRNA and α-BGT substantially antagonized the upregulation of IDO1 by NNK in both cell and mouse models. Treatment of the cells with si*CHRNA7* also suppressed NNK-induced Trp reduction, Kyn elevation, and Trp/Kyn ratio decrease. Moreover, α7nAChR was required for c-Jun activation triggered by NNK. These results suggest that α7nAChR is critical for NNK-related metabolic reprogramming and lung carcinogenesis and represents a potential drug target. Previous studies showed that nicotine binds α7nAChR with a weaker affinity,^51^ providing a reasonable explanation for its weaker effect on reducing Trp/Kyn ratios in 16HBE, A549, EPLC-32M, and LLC cells, compared with that of NNK. However, the mechanisms underlying c-Jun activation by the NNK-α7nAChR cascade remain to be investigated. In addition, α7nAChR is also expressed by CD4^+^ T cells and mediates nicotine-dependent upregulation of Tregs,^52^ further suggesting the critical roles of tobacco smoke in shaping the immunosuppressive microenvironment in lung tumorigenesis.

IDO1 is a promising target for anticancer drug development.^10^ In NSCLC, IDO1 is positive in nearly 60% (229/388) of patients,^29^ and its overexpression is associated with resistance to chemotherapy.^53^ Meanwhile, coexpression of IDO1 and PD-L1 is detected in 28% (109/388) of NSCLC cases^29^ and is associated with aggressive features of LUAD.^54^ IDO1 deficiency reduces lung tumor burden and improves survival in KRAS-induced lung carcinoma and breast carcinoma-derived pulmonary metastasis models,^55^ and inhibition of IDO1 may overcome resistance to anti-PD1 treatment by blocking MDSCs.^56^ However, IDO1 inhibitors did not show satisfactory efficacy in cancers, including NSCLC,^12^ suggesting that biomarkers for patient stratification are required to optimize IDO1 in treating lung cancer. We found that smokers with NSCLC with a lower Trp/Kyn ratio were associated with a worse prognosis, and smokers with a higher Trp/Kyn ratio showed a better response to pembrolizumab. Consistently, smokers with NSCLC with higher IDO1 expression responded poorly to pembrolizumab. Therefore, IDO1 might be a potential response marker of immune checkpoint inhibitors in lung cancer patients with a smoking history. Our results also suggested that IDO1-high smoker patients may benefit from combinatory regimens containing antibodies against PD1/PD-L1 and IDO1 inhibitors. Clinical trials are suggested to test the efficacy of combinatory therapy of IDO1 inhibitor and PD1/PD-L1 blockade in treating smoker patients with high level of IDO1, which may probably revive IDO1 inhibitor for cancers.

## Materials and methods

### Patient samples

The study was approved by the research ethics committee of our hospital and was conducted in accordance with the tenets of the Declaration of Helsinki. The diagnosis of NSCLC was confirmed by at least two pathologists, and all samples were harvested with written informed consent. Tumor specimens were harvested at the time of surgery and were quickly frozen in liquid nitrogen, blood samples were obtained at the time of diagnosis, and plasma was collected after centrifugation at 4 °C and stored at −80 °C. The baseline demographic characteristics of the patients are listed in Tables 1, 2 and Supplementary Table 1.

### Animals

The animal studies were approved by the Institutional Review Board of our hospital with protocols approved by the Animal Ethics Committee of our hospital. The A/J mice (5- to 6-week old, female) were obtained from the Jackson Laboratory (Bar Harbor, Maine, USA). OT-1 transgenic mice were a generous gift from Professor Bo Huang of the Institute of Basic Medical Sciences, Chinese Academy of Medical Sciences. The A/J mice received gavage administration of NNK at a dose of 50 mg/kg twice a week for 5 weeks, the IDO1 inhibitor 1-MT at 50 mg/kg, or the α7nAChR inhibitor α-BGT at 5 mg/kg, and control mice were treated with saline. In other experiments, the A/J mice inside in a perspex box were exposed to cigarette smoke produced by a smoke generator (Buxco, NC, USA), at a frequency of 12 cigarettes per day 5 days per week for 24 weeks. Control mice were exposed to normal air.

### Cigarette smoke extract and reagents

CSE was prepared by a modified method of Carp and Janoff.^57^ Briefly, two cigarettes without filters were combusted with a smoke generator, the smoke was bubbled through 50 ml serum-free media that were adjusted to pH 7.4 and filtered through a 0.22-μm pore filter to remove large particles and bacteria. The media were considered as 100% and diluted with medium before use.

Benzo[a]anthracene (BaA, Cat: B2209), benzo(a)pyrene (BaP, Cat: B1760), benzyl 4-hydroxybenzoate (BzP, Cat: 380709), benzo[k]fluoranthene (BkF, Cat: 392251), dibenzo[a,h]anthracene (DBA, Cat: 48574), 1-Methyl-tryptophan (1-MT, Cat:447439), Kynurenine (Kyn, Cat: K8625), Tryptophan (Trp, Cat: 93659), α-Bungarotoxin (α-BGT, Cat: 203980) and Ovalbumin (257-264) (OVA, Cat: S7951) were obtained from Sigma-Aldrich, St. Louis, MO, USA. NNK (Cat: SY062201) was obtained from J&K Scientific, Beijing, China. Nicotine (Cat: 440649) was obtained from Absin Bioscience, Shanghai, China. Cadmium oxide (CdO, Cat: M04663), 4-Aminobiphenyl (4-ABP, Cat: M29751) and 1,3-Butadiene (BD, Cat: M5512) were obtained from Meryer Technology, Shanghai, China.

4-ABP, BaA, BaP, BD, BkF, BzP, CdO, and DBA were dissolved in DMSO to a stock concentration of 25 mM and stored at −20 °C, and were diluted with culture medium when used for cell treatment. 1-MT was dissolved in DMSO to a concentration of 2.5 mM and stored at −20 °C. Kyn, Trp, α-BGT, OVA, NNK, and nicotine were dissolved in sterile water to a concentration of 25 mM and stored at −20 °C, and diluted with culture medium when used for cell treatment.

### Cell culture and RNA extraction

The normal human bronchial epithelial cell line 16HBE, human LUAD cell line A549, human LUSC cell line EPLC-32M and mouse Lewis lung carcinoma cell line LLC were cultured in RPMI 1640 or DMEM supplemented with 10% fetal bovine serum (FBS; Gibco, Grand Island, NY, USA), respectively, and treated with CSE or compounds as indicated. Total RNA of the cells was harvested with TRIzol reagent (Invitrogen, Frederick, MD, USA), and the expression of genes of interest was evaluated by qRT-PCR using the primers listed in Supplementary Table 5.

### Measurement of Trp and Kyn concentrations

The concentrations of Trp and Kyn in the plasma and supernatant of the cell culture were determined by HPLC. Protein in samples was dislodged by trichloroacetic acid. Purified samples were separated on reversed-phase C18 material using 0.015 M sodium acetate buffer (pH = 6.4) with 2.7% acetonitrile. Trp and Kyn were measured by UV absorption at 279 and 360 nm, respectively, and the Trp/Kyn ratio was calculated by dividing Trp concentrations (μmol/L) by Kyn concentrations (μmol/L).

### Immunohistochemistry assay

Immunohistochemistry (IHC) assays were performed in lung tissue samples isolated from patients and mice. Tumor tissues were fixed with formalin, embedded in paraffin, and cut into 3–5 μm slides. The slides were then deparaffinized with xylene and graded alcohol, and underwent a heat-induced epitope retrieval step in citrate buffer solution. The slides were blocked with 5% BSA, incubated with an anti-IDO1 antibody (ab106134, Abcam, USA; 1:50), and then incubated with an HRP-labeled goat anti-mouse IgG antibody. Immunoreactions were evaluated with the 3,3′-diaminobenzidine (from Zhongshan Golden Bridge Biotechnology Co., Ltd., Beijing, China) and hematoxylin. The percentage of staining-positive cells (RP) was evaluated, the staining intensity (SI) was determined, and the immunoreactivity score (IRS) was calculated as IRS (0–12) = RP (0–4) × SI (0–3).

### Chromatin immunoprecipitation (ChIP)

The cells (2 × 10^7^) were fixed with 1% formaldehyde and then stopped by 0.125 M glycine. The protein-bound chromatin was sheared into small fragments by sonication, and the specific protein/DNA complexes were immunoprecipitated at 4 °C overnight with an anti-c-Jun antibody (#9165S, Cell Signaling Technology, Danvers, MA, USA; 1:100) for ChIP or normal rabbit IgG (#2729, Cell Signaling Technology, USA, 1:100) as a control. Protein A/G PLUS-agarose (#sc-2003, Santa Cruz Biotechnology, Santa Cruz, CA, USA; 1:50) was then supplemented and incubated at 4 °C for 6 h. After immunoprecipitation, DNA was decrosslinked, the proteins were removed by incubation with proteinase K, and the DNA was purified for qRT-PCR using primers listed in Supplementary Table 5.

### Flow cytometry

Mouse lung tissue specimens were cut into 2 mm fragments and digested with collagenase IV (0.3%; Sigma) to obtain separated cells, which were passed through a 200 mm stainless steel wire mesh to obtain a single-cell suspension. The cells were then labeled with indicated markers, sorted by BD Fortessa (LSRFortessa X-20, BD, USA), and analyzed by the FlowJo (BD). Matched isotype controls were used in all experiments.

### T cells and Tregs

Ovalbumin (OVA)-specific CD8^+^ T cells were harvested from the spleen and LNs of C57BL/6/OT-1 transgenic mice by the Moflo (Moflo Astrios EQ, Beckman Coulter, Miami, FL, USA) cell sorting system and an PE-CY7-labeled anti-CD8 antibody. Naïve CD4^+^ T cells were harvested from the spleen and LNs of the C57BL/6 mice using a magnetic cell separation system and a mouse naïve CD4^+^ T-cell Isolation Kit (Miltenyi Biotec, Friedrich, Germany).

The isolated CD8^+^T cells were labeled with CFSE, cultured in RPMI 1640 supplemented with 10% FBS and 1 ng/ml IL-2 (R&D, Minneapolis, MN, USA) and activated with an anti-CD3/CD28 antibody. CD8^+^ T-cell proliferation was assessed by CFSE fluorescence on day 4. LLC-OVA cells were labeled with CFSE and cocultured with CD8^+^ T cells isolated from OT-1 transgenic mice in round-bottom plates, and propidium iodide (PI) was added after 48 h to measure the cytotoxicity.

Naïve CD4^+^ T cells were cultured in medium RPMI 1640 with 10% FBS, 1 ng/ml IL-2 (R&D), and 5 ng/ml TGF-β (R&D) and activated with the anti-CD3/CD28 antibody. The differentiation ratio was assayed by labeling CD25 and Foxp3 in cells and detection by flow cytometry.

### Immunofluorescence microscopy

Cells were grown on cover slides and were fixed with 4% paraformaldehyde, washed with 150 mM glycine in PBS, and permeabilized with 0.3% Triton X-100 in PBS. Cells were blocked in 5% BSA, incubated with the indicated primary antibodies overnight at 4 °C, washed, and then co-incubated with Alexa Flour^®^ 488-labeled secondary antibody (Life Technology, Thermo Fisher Scientifi, Basingstoke, UK). The slides were mounted with antifade medium, observed under a laser scanning confocal microscope (Dragonfly 505, Andor, UK), and cell images were taken and analyzed.

### Plasmids and luciferase assay

The promoter region of *IDO1*, −1800 to −30 bases upstream of the initiating ATG codon, was amplified by PCR (see Supplementary Table 5 for the primer sequences) and cloned into the pGL3-BASIC plasmid (E1751, Promega, Madison, WI, USA) using restriction endonucleases Xho I (1094AH, TaKaRa, Tokyo, Japan) and Hind III (1060AH, TaKaRa). The cells were transfected with plasmids containing *IDO1* promoter-driven luciferase and small interfering RNAs (siRNAs) (Supplementary Table 5). Luciferase activity was measured using the Dual luciferase reporter assay system (Promega, Madison, WI, USA).

### Western blot

Cells and tissues were lysed with RIPA buffer, protein were extracted and quantitated, separated by 10% sodium dodecyl sulfate-polyacrylamide gel electrophoresis (SDS-PAGE), and then transferred onto a PVDF membrane. The protein containing membrane was washed, incubated with the indicated primary antibodies, washed, and further incubated with secondary antibodies. After washing with PBS, the proteins on the membrane was then detected by electrochemiluminescence (ECL) in a Luminescent Image Analyzer LSA 4000 (GE, Fairfield, CO, USA). The antibodies used in this study included rabbit anti-β-Actin (AC026, Abclonal, USA; 1:50000), rabbit anti-c-Jun (#9165S, Cell Signaling Technology, USA, 1:500), rabbit anti-p-c-Jun (#9261S, Cell Signaling Technology, 1:500), rabbit anti-α7nAChR (#ab216485, Abcam, Cambridge, MA, USA; 1:500) and rabbit anti-IDO1 (ab106134, Abcam; 1:500) antibodies.

### Statistical analysis

All results are expressed as mean ± SD of three or more biologically independent experiments. Survival curve was estimated by the Kaplan–Meier method and log-rank test. The correlation between immunoreactivity score of IDO1 and Try/Kyn ratio was evaluated by Pearson’s correlation. The strength of the correlation was evaluated by the following standard: 0.00–0.29 as weak, 0.30–0.49 as moderate, 0.50–0.79 as strong, and 0.80–1.0 as very strong. The statistical analyses were conducted using a software, GraphPad Prism 5 (GraphPad Software, Inc., La Jolla, CA, USA). Statistically significant differences were determined by Students *t* test or Fisher’s exact test. *P* values <0.05 were considered statistically significant.

## Supplementary information

Supplementary information

Supplementary Table 1

## Data Availability

All data supporting this paper are presented within the paper and/or the Supplementary Materials. The original data sets are also available from the corresponding author upon reasonable request.
