# Supplementary material for: Tobacco carcinogen induces tryptophan metabolism and immune suppression via induction of indoleamine 2,3-dioxygenase 1
Source: Signal Transduct Target Ther. 2022 Sep 7;7:311. doi: 10.1038/s41392-022-01127-3 (PMC9448807; doi:10.1038/s41392-022-01127-3)
Supplement: Supplementary file 1 — Supplementary information [file 41392_2022_1127_MOESM1_ESM.docx]

**Supplementary materials for**

**Tobacco carcinogen induces tryptophan metabolism and immune suppression via induction of indoleamine 2,3-dioxygenase 1**

Fan Liang^1,2^*, Gui-Zhen Wang^1^*, Yan Wang^3^*, Ya-Ning Yang^3^, Zhe-Sheng Wen^4^, Dong-Ni Chen^4^, Wen-Feng Fang^4^, Bin Zhang^5^, Lu Yang^3,6^, Chen Zhang^2^, Si-Chong Han^1^, Fu-Ying Yang^1^, Di Wang^1^, Li-Jun Liang^1^, Zheng Wang^1^, Yong Zhao^2^, Chang-Li Wang^5^, Li Zhang^4^, Guang-Biao Zhou^1*^

This file includes Supplementary Tables 2 – 5 and Supplementary Figures 1 – 3.

**Supplementary Table 2.** Baseline demographic characteristics of the 72 smokers with NSCLC.

| **Variable** | **No. of cases (%)** | **Trp/Kyn ratio** | | | |
| --- | --- | --- | --- | --- | --- |
|  |  | **Median (range)** | **High, n (%)** | **Low, n (%)** | ***P* values*** |
| **Total** | 72 | 25.86 (7.36-43.34) | 26 (36.1) | 46 (63.9) |  |
| **Age** |  |  |  |  | 0.141 |
| < 60 | 35 (48.6) | 26.39 (7.96-43.34) | 16 (45.7) | 19 (54.3) |  |
| ≥ 60 | 37 (51.4) | 25.79 (7.36-40.78) | 10 (27) | 27 (73) |  |
| **Gender** |  |  |  |  | 0.352 |
| Male | 59 (81.9) | 25.79 (7.36-43.34) | 23 (39) | 36 (61) |  |
| Female | 13 (18.1) | 26.8 (11.65-33.67) | 3 (23.1) | 10 (76.9) |  |
| **Histology**  LUAD  LUSC  **Stage** | 34 (47.2)  38 (52.8) | 26.21 (7.36-43.34)  25.86 (9.62-41.88) | 12 (35.3)  14 (36.8) | 22 (64.7)  24 (63.2) | 1  0.128 |
| I- II | 24 (33.3) | 28.53 (16.08-41.88) | 12 (50) | 12 (50) |  |
| III-IV  Unknown | 33 (45.8)  15 (20.8) | 22.08 (7.36-43.34)  26.99 (15.44-39.69) | 8 (24.2)  6 (40) | 25 (75.8)  9 (60) |  |

**P* values were calculated using a two-sided Fisher’s exact test.

**Supplementary Table 3.** Baseline demographic characteristics of the 70 nonsmokers with NSCLC.

**P* values were calculated using a two-sided Fisher’s exact test.

| **Variable** | **No. of cases (%)** | **Trp/Kyn ratio** | | | |
| --- | --- | --- | --- | --- | --- |
|  |  | **Median (range)** | **High, n (%)** | **Low, n (%)** | ***P* values*** |
| **Total** | 70 | 32.46 (15.94-72.85) | 45 (64.3) | 25 (35.7) |  |
| **Age** |  |  |  |  | 0.095 |
| < 60 | 36 (51.4) | 34.84 (15.94-72.85) | 27 (75) | 9 (25) |  |
| ≥ 60  Unknown | 33 (47.1)  1 (1.4) | 29.42 (16.81-59.45)  31.74 (31.74-31.74) | 17 (51.5)  1 (100) | 16 (48.5)  0 (0) |  |
| **Gender** |  |  |  |  | 0.528 |
| Male | 18 (25.7) | 30.42 (18.81-59.45) | 10 (55.6) | 8 (44.4) |  |
| Female  Unknown | 51 (72.9)  1 (1.4) | 33.02 (15.94-72.85)  31.74 (31.74-31.74) | 34 (66.7)  1 (100) | 17 (33.3)  0 (0) |  |
| **Histology**  LUAD  LUSC  Unknown  **Stage** | 60 (85.7)  9 (12.9)  1 (1.4) | 33.37 (15.94-72.85)  27.37 (16.81-59.45)  31.74 (31.74-31.74) | 40 (66.7)  4 (44.4)  1 (100) | 20 (33.3)  5 (55.6)  0 (0) | 0.325  0.131 |
| I- II | 25 (35.7) | 34.04 (16.92-72.85) | 18 (72) | 7 (28) |  |
| III-IV  Unknown | 31 (44.3)  14 (20) | 28.78 (15.94-62.06)  33.33 (21.18-68.69) | 16 (51.6)  11 (78.6) | 15 (48.4)  3 (21.4) |  |

**Supplementary Table 4.** Predictive transcription factors that can regulate *IDO1* in human and mouse.

| **Human** | **Mouse** |
| --- | --- |
| C/EBPbeta | C/EBPalpha |
| c-Jun | c-Jun |
| ER-alpha | c-Fos |
| FOXP3 | C/EBPbeta |
| HNF-3alpha | HOXA5 |
| IRF-2 | JunD |
| GR | NF-1 |
| GR-alpha | USF-1 |
| GR-beta | YY1 |
| p53 |  |
| Pax-5 |  |
| PR A |  |
| PRB |  |
| STAT4 |  |
| TCF-4E |  |
| TFII-I |  |
| TFIID |  |
| XBP-1 |  |

**Supplementary Table 5. Sequence of primers and siRNAs used in the study.**

| **Target** | | **Forward primer (5’-3’)** | **Reverse primer (5’-3’)** | |  |
| --- | --- | --- | --- | --- | --- |
| **ChIP** | |  |  | |  |
| **Human** | |  |  | |  |
| *IDO1* | | ATCCTGGGACAGAATCATCT | TTGAGCATTAGAAAATATGTGGTTA | |  |
| **Mouse** | |  |  | |  |
| *IDO1* | | CAATGGGCCTCTCTTTCCAGT | ATTGGATGGATCACAGGGCT | |  |
| **siRNA** | |  |  | |  |
| **Human** | |  |  | |  |
| siNC | | UUCUCCGAACGUGUCACGU | ACGUGACACGUUCGGAGAA | |  |
| si*IDO1* | | GAACGGGACACUUUGCUAA | UUAGCAAAGUGUCCCGUUC | |  |
| si*CHRNA7* | | GGACAGAUCACUAUUUACA | UGUAAAUAGUGAUCUGUCC | |  |
| si*CHRNA5* | | CCCGCAAACUACAAAAGUU | AACUUUUGUAGUUUGCGGG | |  |
| si*Jun* | | GGAAGCUGGAGAGAAUCGC | GCGAUUCUCUCCAGCUUCC | |  |
| **Mouse** | |  |  | |  |
| siNC | | GCCAUGACCACAUGGACGA | CGGUACUGGUGUACCUGCU | |  |
| si*IDO1* | | GCUUCGAGAAGAAGUUGAA | UUCAACUUCUUCUCGAAGC | |  |
| si*CHRNA7* | | GCAGAUAUCAGCAGCUAUA | UAUAGCUGCUGAUAUCUGC | |  |
| si*Jun* | | CAGCUUCCUGCCUUUGUAA | UUACAAAGGCAGGAAGCUG | |  |
| **qPCR**  **Human** | |  |  | |  |
| *GAPDH* | | GGAGCGAGATCCCTCCAAAAT | GGCTGTTGTCATACTTCTCATGG | |  |
| *IDO1* | | TGGGGCAAAGGTCATGGAG | TTTCTTGGAGAGTTGGCAGTAAG | |  |
| *IDO2* | | CCACAGACCGAATGTGAAGAC | TGTTGGCAATTTCCATCCAAGG | |  |
| *TDO* | | AAGGTTGTTTCTCGGATGCAC | TGTCATCGTCTCCAGAATGGAA | |  |
| *TPH1* | | ACGTCGAAAGTATTTTGCGGA | ACGGTTCCCCAGGTCTTAATC | |  |
| *CHRNA1* | | GAGTCTAACAATGCGGCG | TTTCTGCTCATCCTTGCTG | |  |
| *CHRNA2* | | TCCTGTGTTCCTGTCCTTCA | GGTCCTCAGTCTCGGTATG | |  |
| *CHRNA3* | | GAGAAGGTGACCCTGTG | GTGATGACGATGGACAAGGTTA | |  |
| *CHRNA4* | | GTTGGCGTATTTGCGTC | GAGAGGTCAATCCACGG | |  |
| *CHRNA5* | | ACGCTTCCCAAACTGCT | CTTCAACAACCTCACGG | |  |
| *CHRNA6* | ACGCTTTGTATTTCAGTCCT | | | GGGTGCGGTAGTGTATG | |
| *CHRNA7* | CGGCAAGAGGAGTGAAA | | | GAACACCAGCAGGGCGA | |
| *CHRNA9* | AGGGTGGTCATCCTGAAAT | | | AGGTCTTTGTTCCTGGC | |
| *CHRNA10* | CACAAGGGAGCACTCAT | | | CCAATACCCAGCACAAAC | |
| **Mouse** |  | | |  | |
| *GAPDH* | AGGTCGGTGTGAACGGATTTG | | | GGGGTCGTTGATGGCAACA | |
| *IDO1* | CAAAGCAATCCCCACTGTATCC | | | ACAAAGTCACGCATCCTCTTAAA | |
| *IDO2* | TCAAAGTCAGAGCATGACGCT | | | GGCGGTTCTCGATTAAGTGAG | |
| *TDO* | AATCCATGACGAGCACCTATTCA | | | GCTATCACCTTGAGCATGTTCCT | |
| *TPH1* | AACAAAGACCATTCCTCCGAAAG | | | TGTAACAGGCTCACATGATTCTC | |
| *CHRNA1* | CTCTCGACTGTTCTCCTGCTG | | | GTAGACCCACGGTGACTTGTA | |
| *CHRNA2* | TTATCTCTGGTGTCTGCTTCTGA | | | CCCAGCGATTGTAGCCTCC | |
| *CHRNA3* | TCCAGTTTGAGGTGTCTATGTCT | | | TGGTAGTCAGAGGGTTTCCATTT | |
| *CHRNA4* | CTAGCAGCCACATAGAGACCC | | | GACAAGCCAAAGCGGACAAG | |
| *CHRNA5* | ATCCTCTGCTGCAAAACATGA | | | TCCACGTCCACTAACTGAGAT | |
| *CHRNA6* | TAAAGGCAGTACAGGCTGTGA | | | AAAATGCACCGTGACGGGAT | |
| *CHRNA7* | CACATTCCACACCAACGTCTT | | | AAAAGGGAACCAGCGTACATC | |
| *CHRNA9* | GGAACCAGGTGGACATATTCAAT | | | GCAGCCGTAGGAGATGACG | |
| *CHRNA10* | GCTCACAAGCTGTTTCGTGAC | | | ACTTGGTTCCGTTCATCCATATC | |
| **Promoter cloning** | | | | | |
| **pGL3-*IDO1*** | CTAGCCCGGGCTCGAGTTGGTTCTGCAGTCAAGGGTAGG | | | CCGGAATGCCAAGCTTCAGTGTCTGAAGAGTTTTCAGAGC | |

**Supplementary figures and legends**


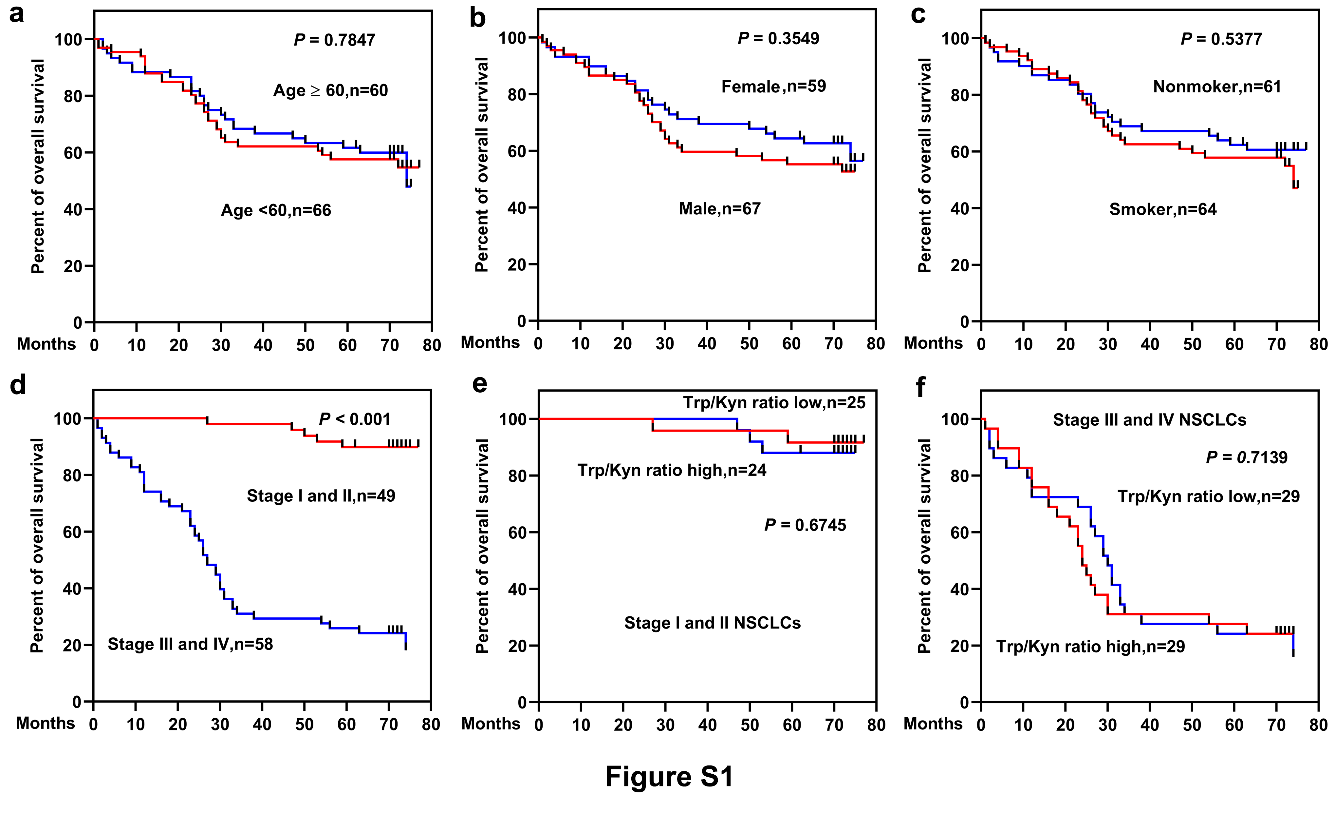


**Supplementary Fig. 1. Overall survival of patients with NSCLC.** The potential effects of the age (a), gender (b), smoking status (c) and disease stage (d) on the prognosis of the patients are shown. The potential effects of the Trp/Kyn ratios on the outcome of NSCLC patients at Stage I/II (e) and Stage III/IV are also shown.


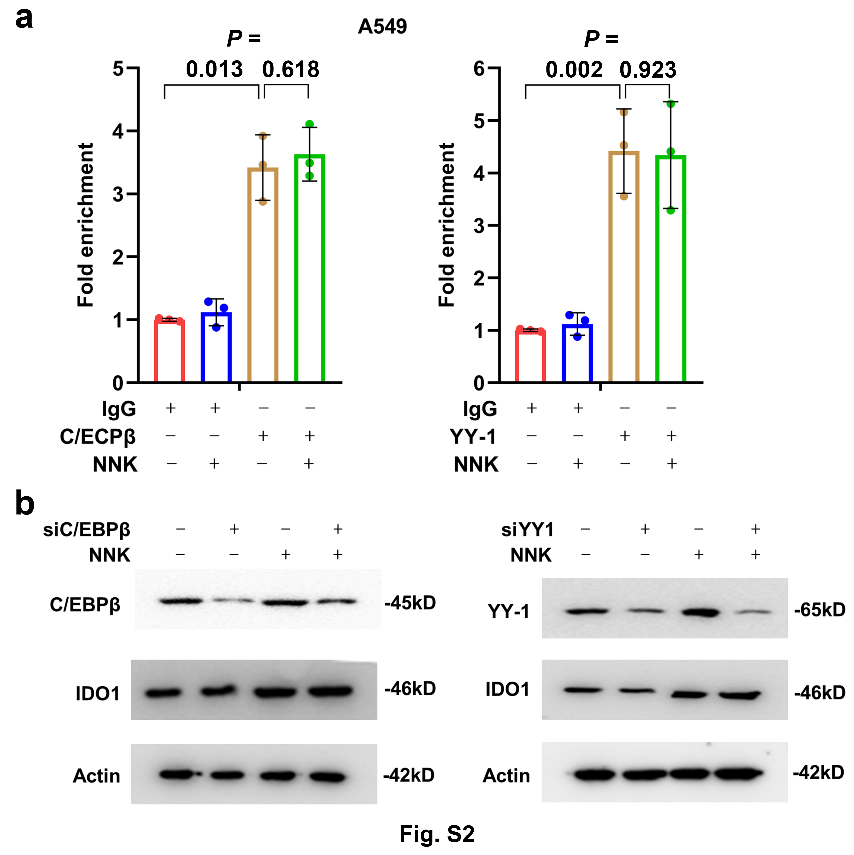


**Supplementary Fig. 2. The effects of C/EBPβ and YY1 on NNK-induced upregulation of IDO1.** (a) A549 cells were treated with or without NNK and lysed, chromatin immunoprecipitation assays were performed using IgG or an anti-C/EBPβ (left) or an anti-YY1 (right) antibody, and the precipitated DNA was used to detect the expression of *IDO1* by qRT-PCR. (b) A549 cells were transfected with siC/EBPβ (left) or siYY1 (right), treated with 25 μM NNK for 72 hours, lysed and subjected to Western blotting using the indicated antibodies.


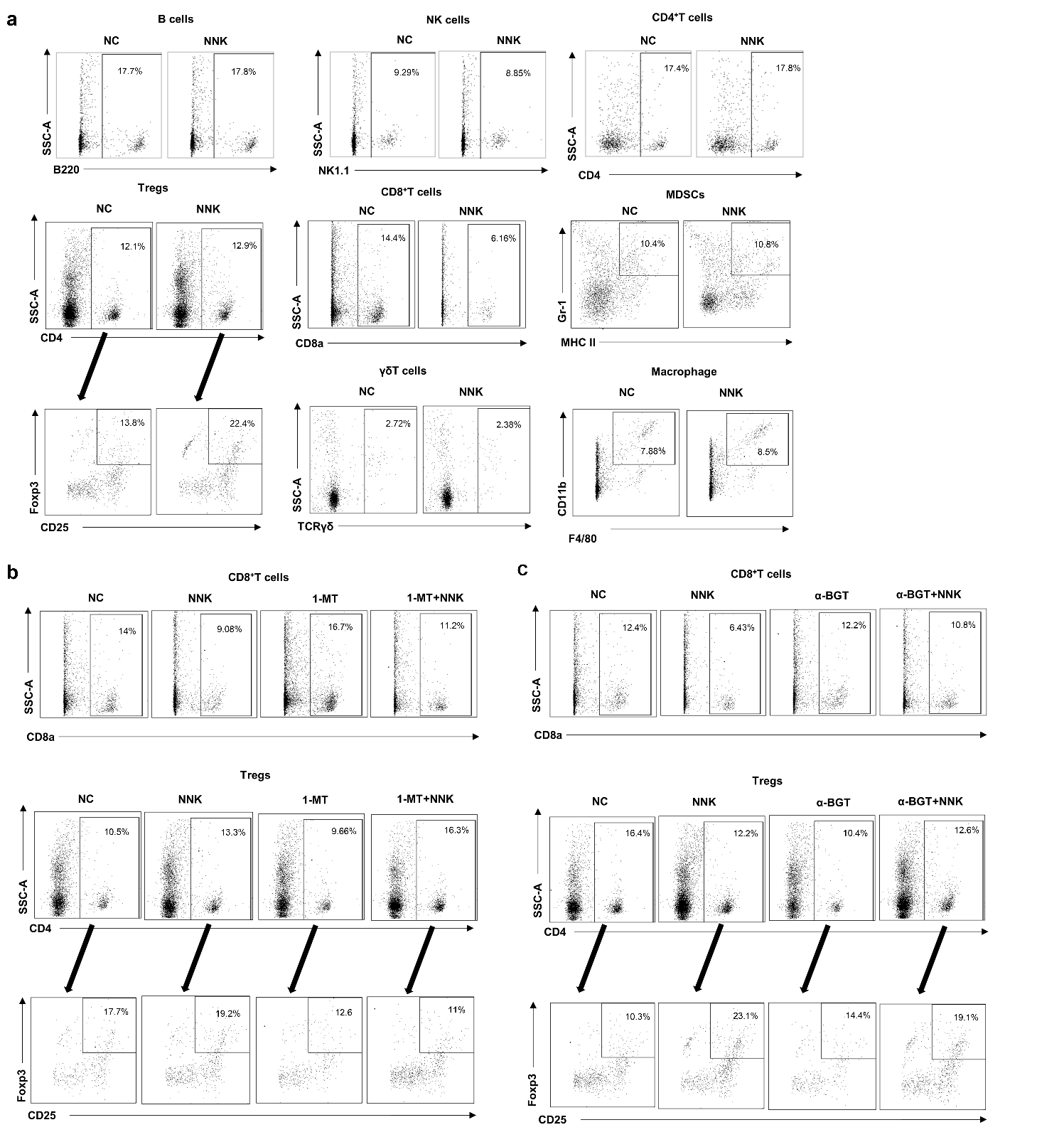


**Supplementary Fig. 3. Representative FACS plots of immune cells in mouse lung.** (a) FACS plots of B cells, NK cells, CD4^+^T cells, Tregs, CD8^+^T cells, MDSCs, γδT cells and macrophage in lung tissues of mice treated with NNK at 50 mg/kg/day for 90 days. (b) FACS plots of Tregs and CD8^+^T cells in lung tissues of mice treated with NNK and 1-MT for 90 days. (c) FACS plots of Tregs and CD8^+^T cells in lung tissues of mice treated with NNK and α-BGT for 90 days.
